# Depression During COVID-19 Quarantine in South Korea: A Propensity Score-Matched Analysis

**DOI:** 10.3389/fpubh.2021.743625

**Published:** 2022-01-27

**Authors:** Yongjoo Kim, Hye-Young Kwon, Seungyoung Lee, Chang-Bo Kim

**Affiliations:** ^1^College of Korean Medicine, Sangji University, Wonju, South Korea; ^2^Division of Biology and Public Health, Mokwon University, Daejeon, South Korea; ^3^College of Nursing, Kyunghee University, Seoul, South Korea; ^4^Seoul Health Foundation, Seoul, South Korea

**Keywords:** COVID-19, quarantine, mental health, psychological impacts, depression, depressive symptoms, depressive disorder, South Korea

## Abstract

**Background:**

Quarantine, a public health measure used to control the coronavirus disease 2019 (COVID-19) pandemic, has been linked to an increased risk of developing adverse psychological sequelae. This study sought to investigate whether quarantining during the COVID-19 pandemic was associated with depression among Koreans.

**Methods:**

Data were obtained from the Seoul COVID-19 Study of Quarantine (SCS-Q) and the 2019 Korea Community Health Survey (KCHS). Using propensity scores estimated based on sociodemographic and health conditions, 919 individuals undergoing quarantine in the SCS-Q were matched with 919 individuals who did not experience quarantine in the 2019 KCHS. Depressive symptoms were measured using the Korean version of the Patient Health Questionnaire-9 (PHQ-9), where major depression is defined as a PHQ-9 score ≥ 10. Logistic regression models were adjusted for sociodemographic and health-related factors.

**Results:**

Depression prevalence was higher in quarantined individuals than in the control group (7.8 vs. 3.8%, *p* < 0.001). Logistic regression analyses revealed that quarantining was associated with higher likelihoods of having major depression [odds ratio (OR) = 2.28, 95% confidence interval (CI): 1.49, 3.51] after adjusting for relevant covariates.

**Limitations:**

Due to the online nature of the SCS-Q, this study included a limited number of elderly participants, limiting the generalizability of the findings to the general Korean population.

**Conclusions:**

The findings suggest that Koreans undergoing COVID-19 quarantine are at higher risk of depression. While further investigation is warranted, public health measures to control infectious disease outbreaks, such as quarantine, would benefit from incorporating strategies to address unintended adverse psychological effects, such as depression.

## Introduction

The novel coronavirus disease 2019 (COVID-19) pandemic is an urgent global public health issue. Quarantine is one of the most commonly used public health measures to address the spread of infectious disease outbreaks, limiting the movement of people who are at high risk of exposure to an etiological agent, even in the absence of clinical symptoms or laboratory results (1). However, due to the unpleasant nature of quarantine stemming from separation and restriction, adverse psychological sequelae are pervasive among individuals who undergo quarantine (2–4).

A growing body of literature has documented that quarantining is associated with elevated levels of psychological distress, including depression, anxiety, and suicidality (4–7). A recent review reported that quarantining due to infectious disease outbreaks can be followed by psychological and environmental stressors such as fears/concerns of infection, loss of social relationships and physical activities, and insufficient supplies and information, which in turn can lead to mental disorders, including depression, anxiety, and post-traumatic stress symptoms (4). In relation to COVID-19 quarantine, generally higher levels of psychological distress symptoms have been reported among individuals undergoing quarantine in China (3, 5), Ireland (8), and Italy (9). Moreover, a Canadian study found that, compared to individuals who did not undergo quarantine, those who experienced quarantine tended to have increased suicidality, self-harm intentions, and other unfavorable mental health outcomes (7). These previous studies necessitate further research to inform effective intervention strategies to address adverse psychological consequences during the implementation of quarantine.

In South Korea, after the first laboratory-confirmed case of COVID-19 was detected in January 2020, the spread of COVID-19 has been relatively successfully controlled to the extent that it does not require a nationwide lockdown (10). Based on the “3T” strategy (Testing-Tracing-Treating), mandatory quarantine has been widely implemented among high-risk populations. That is, individuals who had close contact with those who received positive laboratory test results, even in the absence of clinical symptoms, or individuals who traveled abroad were required to have a 14-day self-quarantine at home or dedicated facilities (10). Previous Korean studies have generally focused on the levels of psychological distress among the general public during the COVID-19 pandemic or individuals infected with COVID-19 (11–13). For instance, Kim and colleagues found higher levels of sleep disturbance and perceived stress among the general public residing where COVID-19 was prevalent (11). However, despite the accumulated number of individuals who experienced or were undergoing quarantine in South Korea and the increasing body of evidence showing the negative psychological impacts of quarantine among other populations, to date, there has been limited evidence to understand whether COVID-19 quarantine leads to an elevated risk of mental disorders among Koreans. Therefore, in this study, we sought to investigate the association between quarantining during the COVID-19 pandemic and depression among Koreans. Based on previous research, we hypothesized that individuals undergoing COVID-19 quarantine would have an elevated risk of depression and higher levels of depressive symptoms than those who did not experience quarantine.

## Methods

### Sample and Data Source

In South Korea, all individuals who enter the country from abroad or make close contact with those affected by COVID-19 are recommended to undergo screening tests at local screening posts. Of those, individuals who receive negative test results are required to conduct self-quarantine in a dedicated facility or at home for 14 days, whereas those who receive a positive test result are transferred to designated hospitals or residential centers for surveillance. In Seoul Metropolitan City, local district governments and public health centers are in charge of the self-quarantine process, including managing screening posts, providing quarantine guidelines and necessary supplies, and monitoring and communicating with those who are quarantined.

The Seoul COVID-19 Study of Quarantine (SCS-Q) was conducted by the Seoul Health Foundation (SHF) in collaboration with the local district public health centers. The SHF is a public institute established by the Seoul Metropolitan City in order to develop and evaluate public health policies and interventions. In collaboration with local public health centers in Seoul, investigators at SHF developed and administered an online survey. All individuals aged 19 years or above who were undergoing self-quarantine at some point from October to November 2020 in Seoul were sampled and invited to participate in the survey. During the survey period, 5,175 individuals underwent self-quarantine. Of those, 1,139 individuals (overall response rate: 22.0%) agreed to participate in the survey and responded accordingly.

The survey questionnaire was composed of three parts: (a) sociodemographic information including age, sex, socioeconomic status, living arrangement, and residing area; (b) evaluation of quarantine-related processes and experiences; and (c) health-related factors such as depression, anxiety, health-related quality of life, self-rated health, and other medical histories.

The Institutional Review Board of Seoul Metropolitan City approved our study (IRB No. 2020-10-0001). We obtained online informed consent from all survey respondents prior to survey participation.

To select a control group, we used data from the Korea Community Health Survey (KCHS), which is a nationally representative study of Korean community-dwelling individuals aged 19 years or older, measuring information on sociodemographic, behavioral, and medical conditions, administered by the Korea Centers for Disease Control and Prevention (14). The KCHS measures information on sociodemographic, behavioral, and medical conditions. Of the individuals included in the 2019 KCHS, we focused on samples from Seoul, including 3,649 individuals.

Based on the information from the two samples (*N* = 1,139 from the SCS-Q and *N* = 3,649 from the 2019 KCHS), we used a propensity score matching method to match individuals from the SCS-Q with individuals from the 2019 KCHS. First, we built logistic regression models to estimate the propensity to be quarantined with respect to age, sex, district of residence, education, employment status, income level, and chronic conditions such as hypertension, which were determined using a stepwise model selection process. Based on the propensity score estimated by the function of the aforementioned independent variables, the samples from the SCS-Q (*N* = 919) were matched to samples from the 2019 KCHS (*N* = 919), including a total of 1,838 individuals, as the final analytic sample of this study.

### Measures

We used the Korean version of the Patient Health Questionnaire-9 (PHQ-9) to assess depressive symptoms. The PHQ-9 is a commonly used validated measure for depression and comprises nine items capturing symptoms of depression, including anhedonia, depressed mood, trouble sleeping, feeling tired, change in appetite, guilt/self-blame, trouble concentrating, feeling restless/slowed down, and suicidal thoughts, over the past 2 weeks (15). Per each item, response options represent the perceived frequency of the depressive symptom specified in each item during the past 2 weeks, including “never (0),” “several days (1),” “more than half of the days (2),” and “almost every day (3),” resulting in a total score ranging from 0 to 27 (15). Previous studies have reported excellent level of internal consistency reliability of PHQ-9 with Cronbach's alpha values ranging from 0.81 to 0.95 among US and Korean samples (16–19). A consistent result was found in our sample with a Cronbach's alpha value of 0.87. A meta-analysis reported that a cut-off of ≥10 is one of the most commonly used thresholds to identify major depressive disorder with a sensitivity of 0.85, and specificity of 0.89, when compared with a structured psychiatric interview (20). The validity and reliability of the Korean translated version of the PHQ-9 have been reported in previous studies (16, 18, 19). The Korean version of the PHQ-9 was administered to the 2019 KCHS cohort. In the SCS-Q, to assess depressive symptoms during quarantine, the timeframe of each item was modified from “over the past 2 weeks” to “during quarantine.”

### Statistical Analysis

For descriptive analyses, we examined means (standard deviations) for continuous variables and frequencies (proportions) for binary/categorical variables among individuals quarantined during the COVID-19 pandemic (from the SCS-Q) and the control group (from the 2019 KCHS study) before and after matching, respectively. We also examined and compared the distribution of PHQ-9 scores, major depression (PHQ-9 score ≥ 10), and mild depression (PHQ-9 score ≥ 5) in quarantined individuals and the control group after matching. We further examined and compared the distribution of major depression according to relevant covariates, including age (19–39, 40–64, 65+), sex, district of residence, education (high school graduate or less vs. some college education or more), employment status (wage worker, employer/self-employed, economically inactive, others), income level (lowest in quartiles vs. higher than lowest), marital status (single, married, divorced/widowed), living arrangement (living alone vs. others), comorbid conditions such as hypertension and diabetes, and self-rated health (good/very good vs. moderate or worse), among quarantined individuals and the control group after matching.

To investigate whether quarantine during the COVID-19 pandemic was associated with an increased likelihood of major depression, we used a logistic regression modeling approach linking major depression with respect to quarantine status and other relevant independent variables based on the matched data. For all statistical analyses, SAS 9.4 (SAS Institute, Inc. Cary, NC, USA) was used.

## Results

### Descriptive Statistics

Table 1 shows the characteristics of the samples before and after matching. Before matching, significant differences were found in the distribution of relevant sociodemographic and health-related factors between those under quarantine during the COVID-19 pandemic (*N* = 1,139 individuals from the SCS-Q) and control groups (*N* = 3,649 individuals residing in the four local districts in Seoul from the 2019 KCHS). However, after the matching procedure based on the aforementioned propensity score method, those under quarantine during the COVID-19 pandemics (N=919 individuals from the SCS-Q) showed nearly identical characteristics, in terms of the relevant sociodemographic and health-related factors, with the control group (*N* = 919 individuals residing in Seoul from the 2019 KCHS).

**Table 1:** Sociodemographic and health-related characteristics of the SCS-Q sample and control group before and after matching.

**Variables**	**Before matching**							**After matching**					
		**Quarantinees during COVID 19 pandemic**		**Control group**		**χ^2^ or *t*-valtest statisticsue^a^**	***P*-value**	**Quarantinees during COVID 19 pandemic**		**Control group**		**χ^2^ or *t*-value^a^**	***P*-value**
		(***N*** = 1,139)		(***N*** = 3,649)				(***N*** = 919)		(***N*** = 919)			
Male, *N*(%)		565	(49.6%)	1,528	(41.9%)	21.08	<0.0001	468	(50.9%)	468	(50.9%)	0.00	NS
Age, Mean(SD)		39.01	(12.54)	52.35	(17.60)	28.27	<0.0001	39.82	(12.16)	40.10	(13.14)	0.49	NS
**Age group**													
	19-39	608	(53.4%)	941	(25.8%)	472.22	<0.0001	472	(51.4%)	466	(50.7%)	0.08	NS
	40-64	508	(44.6%)	1,690	(46.3%)			427	(46.5%)	433	(47.1%)		
	65 and over	23	(2.0%)	1,018	(27.9%)			20	(2.2%)	20	(2.2%)		
Dwelling district						31.42	<0.0001						
	Nowon-gu	330	(29.0%)	912	(25.0%)			256	(27.9%)	258	(28.1%)	0.06	NS
	Sungbuk-gu	341	(29.9%)	917	(25.1%)			276	(30.0%)	276	(30.0%)		
	Eunpyung-gu	261	(22.9%)	910	(24.9%)			208	(22.6%)	204	(22.2%)		
	Yangcheon-gu	207	(18.2%)	910	(24.9%)			179	(19.5%)	181	(19.7%)		
Income						102.44	<0.0001					0.17	NS
	Lowest	138	(12.1%)	146	(4.0%)			26	(2.8%)	29	(3.2%)		
Employment status						268.85	<0.0001					0.07	NS
	Wage worker	625	(54.9%)	1,620	(44.4%)			576	(62.7%)	554	(60.3%)		
	Employer/Self-employed	98	(8.6%)	407	(11.2%)			86	(9.4%)	115	(12.5%)		
	Economically inactive	312	(27.4%)	1,588	(43.5%)			241	(26.2%)	238	(25.9%)		
	Others	104	(9.1%)	34	(0.9%)			13	(1.4%)	12	(1.3%)		
Education						284.19	<0.0001					0.06	NS
	High school or less	254	(22.3%)	1,850	(50.7%)			155	(16.9%)	151	(16.4%)		
	Tertiary education	885	(77.7%)	1,799	(49.3%)			764	(83.1%)	768	(83.6%)		
Predisposing chronic diseases												0.16	NS
	Hypertension	96	(8.4%)	1,017	(27.9%)	183.90	<0.0001	83	(9.0%)	88	(9.6%)		
Propensity scores		0.38	(0.21)	0.19	(0.15)	28.45	<0.0001	0.32	(0.13)	0.32	(0.13)	0.00	NS

a *Test statistics were driven from t tests for continuous variables, McNemar's test for binary variables, and Chi-squared test for categorical variables*.

Table 2 presents the differences in the distribution of depression measures in those under quarantine during the COVID-19 pandemic (*N* = 919) and in the control group (*N* = 919). Overall, individuals undergoing self-quarantine during the COVID-19 pandemic had higher levels of depressive symptoms (mean score 3.38 vs. 2.29, *p* < 0.001) and a higher prevalence of major (7.8 vs. 3.8%, *p* < 0.001) and mild depression (28.1 vs. 16.8%, *p* < 0.001) than those in the control group. Moreover, major depression was more prevalent among women than among men (10.4 vs. 5.3%), the younger age group (10.2% of those aged 19–39 vs. 5.6% of those aged 40+), economically inactive group than wage workers (11.6 vs. 6.3%), and those living alone than those not living alone (14.0 vs. 6.7%).

**Table 2:** Distribution of depression-related measures among quarantined individuals during the COVID-19 pandemic and the control group.

	**Quarantinees during COVID 19 pandemic**		**Control group**		**χ^2^ or *t*-value**	***P*-value**
	**(*****N*** **=** **919)**		**(*****N*** **=** **919)**			
PHQ score		(4.30)	2.29	(3.257)	6.15	<0.0001
	3.38					
Major Depression^a^, *N* (%)	72	(7.8%)	35	(3.8%)	747.56	<0.0001
Mild/Major Depression^b^, *N* (%)	258	(28.1%)	154	(16.8%)	315.40	<0.0001
By sex					0.35	NS
Male	25	(5.3%)	14	(3.0%)		
Female	47	(10.4%)	21	(4.7%)		
By age					14.78	0.0001
19-40	48	(10.2%)	18	(3.9%)		
40-65	24	(5.6%)	17	(3.9%)		
65 and over	0	(0.0%)	0	(0.0%)		
By district					6.97	NS
Nowon-gu	23	(9.0%)	7	(2.7%)		
Sungbuk-gu	13	(4.7%)	12	(4.3%)		
Eunpyung-gu	13	(6.3%)	10	(4.9%)		
Yangcheon-gu	23	(12.8%)	6	(3.3%)		
By income level					45.00	<0.0001
Lowest	2	(7.7%)	10	(34.5%)		
Others	70	(7.8%)	25	(2.8%)		
By employment					6.27	NS
Wage worker	36	(6.3%)	9	(1.6%)		
Employer/Self-employed	5	(5.8%)	4	(3.5%)		
Economically inactive	28	(11.6%)	21	(8.8%)		
Others	3	(23.1%)	1	(8.3%)		
By education					21.28	<0.0001
High School	12	(7.7%)	19	(12.6%)		
Tertiary Education	60	(7.9%)	16	(2.1%)		
By marital status					15.68	<0.0001
Married	33	(6.3%)	11	(2.1%)		
Single	37	(10.2%)	14	(4.1%)		
Divorced/widowed	2	(5.6%)	10	(17.5%)		
By type of household					30.31	<0.0001
living alone	20	(14.0%)	9	(7.4%)		
others	52	(6.7%)	26	(3.3%)		
By Predisposing diseases						
Hypertension	3	(3.6%)	5	(5.7%)	55.35	<0.0001
Diabetes	2	(4.4%)	3	(7.9%)	61.49	<0.0001
By self-rated health					37.10	<0.0001
Good/very good	32	(7.6%)	1	(0.2%)		
Moderate/bad/very bad	40	(8.0%)	34	(6.7%)		

a *Major depression was defined as PHQ-9 score of 10 or above, representing moderate or severe levels of depressive symptoms*.

b *Mild/Major depression was defined as PHQ-9 score of 5 or above, representing mild, moderate, or severe levels of depressive symptoms*.

### Quarantining and Depression

Table 3 demonstrates the findings from the logistic regression models linking quarantine during the COVID-19 pandemic with major depression (defined as a PHQ-9 score of 10 or above, representing moderate to severe level of depressive symptoms) among the study participants (*N* = 3,649). Overall, individuals undergoing quarantine during the COVID-19 pandemic were more likely to have major depression (OR = 2.28, 95% CI: 1.49, 3.51) than those in the control group, after accounting for relevant sociodemographic and health-related factors. We found a similar association (OR = 2.03, 95% CI: 1.61, 2.56) when using more relaxed criteria to define the outcome (mild/major depression, defined as a PHQ-9 score of 5 or above, representing mild, moderate, or severe level of depressive symptoms).

**Table 3:** Association between quarantine during the COVID-19 pandemic and depression among the matched sample (*N* = 1,838).

		**Major depression^a^**			**Mild/major depression^b^**		
**Factors**		**Odds ratio**	**95% confidence limits**		**Odds ratio**	**95% confidence limits**	
Quarantine during pandemic	(Ref = Pre-pandemic)	2.28	1.49	3.51	2.03	1.61	2.56
Sex	(Ref = Male)	1.33	0.86	2.06	1.37	1.08	1.75
Age		0.99	0.97	1.01	0.98	0.97	0.99
Dwelling district	Nowon	1.29	0.73	2.28	0.86	0.63	1.16
(Ref = Seongbuk)	Eunpyeong	1.18	0.64	2.20	0.86	0.62	1.19
	Yangcheon	2.09	1.17	3.73	0.91	0.65	1.27
Income	(Ref = Middle or High)	2.38	1.07	5.28	2.43	1.33	4.43
Education level	(Ref = Tertiary)	1.61	0.98	2.65	1.34	0.98	1.81
Employment status	Employer/self-employed	1.32	0.62	2.82	1.15	0.78	1.71
(Ref = Salaried workers)	Economically inactive	2.28	1.42	3.64	1.18	0.89	1.55
	Others	4.16	1.29	13.40	1.85	0.78	4.38
Marital status	Single	1.18	0.66	2.12	0.98	0.71	1.35
(Ref = Married)	Divorced/widowed	1.92	0.85	4.31	1.79	1.06	3.01
Hypertension	Yes (Ref = No)	0.88	0.37	2.08	1.10	0.70	1.73
Diabetes	Yes (Ref = No)	1.10	0.39	3.11	1.10	0.62	1.96
Self-rated Health State	Moderate/bad/very bad (Ref = good/very good)	1.87	1.21	2.90	1.79	1.41	2.27
Family size		0.83	0.69	0.99	0.94	0.85	1.04

a *Major depression was defined as PHQ-9 score of 10 or above, representing moderate or severe levels of depressive symptoms*.

b *Mild/Major depression was defined as PHQ-9 score of 5 or above, representing mild, moderate, or severe levels of depressive symptoms*.

We also found other factors associated with depression, including sex, income, employment status, marital status, and self-rated health. For instance, women were more likely to have mild/major depression, defined as a PHQ-9 score of 5 or above, than men (OR = 1.37, 95% CI: 1.08, 1.75) after accounting for all other factors. Individuals in the lowest quartile of income were more likely to have mild/major depression (OR = 2.43, 95% CI: 1.33, 4.43) than those with higher incomes. Similarly, individuals with an economically inactive status were associated with a higher likelihood of having major depression, defined as a PHQ-9 score of 10 or above, than salaried workers (OR = 2.28, 95% CI: 1.42, 3.64). In addition, divorced/widowed individuals were more likely to have mild/major depression (OR = 1.79, 95% CI: 1.06, 3.01) than married individuals and those with moderate or worse self-rated health were more likely to have major depression (OR = 1.87, 95% CI: 1.21, 2.90) than those with good or better self-rated health.

In terms of model fit, our primary model with major depression as a dependent variable, compared to our secondary model with mild/major depression as a dependent variable, generally showed more preferable range of Akaike information criterion (AIC, 818.18 for primary model vs. 1864.07 for secondary) and negative log-likelihoods values (−2^*^LogL, 83.52 for primary vs. 130.00 for secondary).

## Discussion

In the present study, we investigated whether self-quarantine during the COVID-19 pandemic was associated with an increased risk of depression among Korean adults. We used a propensity score matching method and found that individuals undergoing quarantine during the COVID-19 pandemic were more likely to have major and mild depression, as well as higher levels of depressive symptoms than those in the control group after accounting for relevant sociodemographic and health-related factors. To the best of our knowledge, this is the first study to document such associations among the Korean population.

Previous studies have demonstrated generally elevated levels of psychological distress in relation to the COVID-19 pandemic across the general population (2, 21–24) and among multiple subgroups, including individuals undergoing quarantine (3, 5), those infected with coronavirus (25, 26), and front-line healthcare workers (2, 24, 27), with some mixed findings (28, 29).

In terms of psychological distress during quarantine, a review study examining the mental health impacts of quarantine from previous infectious disease outbreaks (e.g., severe acute respiratory syndrome, Middle East respiratory syndrome-related coronavirus, Ebola virus, and H1N1) documented that quarantine has detrimental psychological impacts across multiple populations (4). In relation to the COVID-19 outbreak, Daly and colleagues used a nationwide study of Canadian adults conducted in March 2020, approximately 4 weeks after the early phase of the COVID-19 outbreak in Canada, and found that individuals who went through quarantine for any reason were more likely to have suicidal thoughts and intentional self-harm, as well as more unfavorable mental health status overall, than those who did not (7). The findings were more prominent among those quarantined due to the presence of COVID-19 symptoms or contact with someone who had COVID-19 symptoms, whereas the findings were less noticeable for those quarantined due to recent travel (7). Similarly, high levels of psychological distress (e.g., depressive/anxiety symptoms) have been reported among quarantined populations in China (3, 5), Ireland (8), and Italy (9).

More broadly, in terms of psychological distress among the general population, Ettman and colleagues examined the prevalence of depression measured using the PHQ-9 before and during the COVID-19 pandemic by using nationally representative samples of US adults and found that the prevalence was higher during the COVID-19 pandemic (March-April 2020) than before (2017–2018) for all categories of depression (mild/moderate/moderately severe/severe) (22). Similar findings have been reported for anxiety in the US population (23). Pierce et al. revealed that the prevalence of clinically significant psychological distress symptoms, measured using the 12-item General Health Questionnaire (GHQ-12) was higher during the COVID-19 outbreak (April 2020) than before (2018–2019) among the UK general population, which was confirmed by the significant within-individual increase in GHQ-12 scores based on a nationally representative cohort study (30). Peng et al. demonstrated that, among 2,726 individuals aged 18–70 years who underwent 14 days of quarantine during the COVID-19 pandemic in Shenzhen City, China, the prevalence of depression was 6.2%; the association was more apparent among those who were younger, unmarried, and with lower levels of education (3). Studies have reported that the elevated psychological distress symptoms during the COVID-19 pandemics were more pronounced among women (vs. men), younger age groups (e.g., ≤ 40 vs. 40+ years), those with predisposing chronic physical/psychiatric conditions, those unemployed (vs. employed), and those who have greater exposure to media sources and social media (31, 32).

Moreover, Ma et al. found that, among 770 clinically stable patients with COVID-19 in China, more than 40% exhibited clinically relevant depression symptoms defined as having a PHQ-9 score ≥ 5, whereby the pattern was more pronounced among women (vs. men), those with family member(s) infected with COVID-19 (vs. those without), and those with severe COVID-19 infection (vs. mild/moderate infection) (26).

The findings of our study replicate and extend the prior literature linking quarantining and elevated psychological distress in the Korean population. Our findings are generally consistent with previous evidence showing more unfavorable mental health outcomes, including depression and anxiety, among individuals in North America (7), Asia (3, 5), and European countries (8, 9) quarantined due to the COVID-19 pandemic. More broadly, our findings align with previous literature documenting negative mental health outcomes during the COVID-19 pandemic among the general population (2, 21–24) and other subgroups, including individuals with suspected or laboratory-confirmed COVID-19 (25, 26) and healthcare workers (2, 24, 27). Consistent with previous studies, we also found that depression was more prevalent in women (vs. men), younger individuals (19–39 years vs. 40+), and those living alone (vs. not) during the COVID-19 quarantine.

Potential mechanisms linking COVID-19 quarantine and depression may include elevated levels of negative emotions, such as fear, concerns, frustration, and loneliness. Quarantining due to close contact with an infected individual may cause fear and concerns of infection (4, 7). Moreover, quarantining can lead to limited social relationships, physical activities, and elevated social isolation and loneliness. Insufficient provision of basic supplies and necessary information can also lead to increased psychological burden during quarantine (4, 7). Further prospective investigations are warranted to understand the mechanisms through which quarantine leads to negative psychological consequences.

### Strengths and Limitations

The findings of our study should be interpreted in consideration of the following limitations. First, our study is susceptible to potential selection bias due to the online survey procedure, whereby elderly individuals under quarantine were less likely to participate in the SCS-Q, generally as a result of limited access to the online survey. Together with the fact that our survey was conducted in Seoul Metropolitan City, our findings may not be generalizable to the general Korean population. The control group was selected from participants of the 2019 KCHS before the COVID-19 pandemic. Therefore, the observed association between quarantine during the COVID-19 pandemic and depression may reflect the potentially negative psychological impacts of the COVID-19 pandemic, as well as the impacts of quarantine. However, we were not able to decompose such impacts, warranting further investigation.

Nevertheless, our study has several strengths. Despite the increasing number of individuals who experienced quarantine during the COVID-19 pandemic, to the best of our knowledge, there has been limited evidence regarding the impacts of quarantine on mental health among Koreans. We used a propensity score matching method, through which individuals undergoing quarantine were compared to those who did not experience quarantine but had similar characteristics in terms of sociodemographics and health conditions.

## Conclusions

The findings of our study replicate and extend the findings of previous studies linking quarantine and depression in Korean populations. Our findings suggest that Korean adults who underwent self-quarantine during the COVID-19 pandemic may be at higher risk of developing depression regardless of age, sex, socioeconomic status, living arrangements, and health conditions. Our findings indicate that effective strategies should be developed to prevent and address the psychiatric burden among individuals undergoing quarantine. Specifically, recent studies have emphasized the urgent needs to develop and implement sufficient training and supportive resources to address negative psychological outcomes among quarantine hotel workers during the pandemic (33–35). Similarly, effective quarantine strategies for the general population would benefit from developing and disseminating an innovative virtual platform through which educational programs, coping and counseling sessions, and peer-group support communities can be provided to those undergoing quarantine during the pandemic.

## Data Availability Statement

The raw data supporting the conclusions of this article will be made available by the authors, without undue reservation.

## Ethics Statement

The studies involving human participants were reviewed and approved by the Institutional Review Board of Seoul Metropolitan City approved our study (IRB No. 2020-10-0001). The patients/participants provided their written informed consent to participate in this study.

## Author Contributions

HYK formulated the research question, designed the study, conducted the analysis, and interpreted the results. YK drafted the manuscript. HYK, SL, and CBK reviewed and revised the manuscript. All authors contributed to the acquisition of data, approved the final version of the manuscript, and consented to its publication.

## Conflict of Interest

The authors declare that the research was conducted in the absence of any commercial or financial relationships that could be construed as a potential conflict of interest.

## Publisher's Note

All claims expressed in this article are solely those of the authors and do not necessarily represent those of their affiliated organizations, or those of the publisher, the editors and the reviewers. Any product that may be evaluated in this article, or claim that may be made by its manufacturer, is not guaranteed or endorsed by the publisher.
